# Protective effect of spore oil-functionalized nano-selenium system on cisplatin-induced nephrotoxicity by regulating oxidative stress-mediated pathways and activating immune response

**DOI:** 10.1186/s12951-022-01754-6

**Published:** 2023-02-09

**Authors:** Chaofan Liu, Sajin Zhou, Haoqiang Lai, Lei Shi, Weibin Bai, Xiaoling Li

**Affiliations:** 1 Institute of Food Safety and Nutrition, Jinan University, Guangzhou, 510632 People’s Republic of China; 2 Guangdong Engineering Technology Center of Molecular Rapid Detection for Food Safety, Jinan University, Guangzhou, 510632 People’s Republic of China; 3 The First Affiliated Hospital of Jinan University, Guangzhou, 510632 People’s Republic of China; 4 Department of Chemistry, Jinan University, Guangzhou, 510632 People’s Republic of China

## Abstract

In clinical practice, cisplatin is the most commonly used chemotherapy drug to treat a range of malignancies. Severe ROS-regulated nephrotoxicity, however, restricts its applicability. Currently, the main mechanisms leading to cisplatin-induced nephrotoxicity in clinical settings involve hydration or diuresis. However, not all patients can be treated with massive hydration or diuretics. Therefore, it is crucial to develop a treatment modality that can effectively reduce nephrotoxicity through a foodborne route. Selenium has been reported to have strong antioxidant as well as anticancer effects when administered as spore oil. Herein, we established cellular and animal models of cisplatin-induced nephrotoxicity and synthesized spore oil-functionalized nano-selenium (GLSO@SeNPs). We found that GLSO@SeNPs inhibit the mitochondrial apoptotic pathway by maintaining oxidative homeostasis and regulating related signaling pathways (the MAPK, caspase, and AKT signaling pathways). In vivo, GLSO@SeNPs could effectively improve cisplatin-induced renal impairment, effectively maintaining oxidative homeostasis in renal tissues and thus inhibiting the process of renal injury. In addition, GLSO@SeNPs were converted into selenocysteine (SeCys2), which may exert protective effects. Furthermore, GLSO@SeNPs could effectively modulate the ratio of immune cells in kidneys and spleen, reducing the proportions of CD3^+^CD4^+^ T cells, CD3^+^CD8^+^ T cells, and M1 phenotype macrophages and increasing the proportion of anti-inflammatory regulatory T cells. In summary, in this study, we synthesized food-derived spore oil-functionalized nanomaterials, and we explored the mechanisms by which GLSO@SeNPs inhibit cisplatin-induced nephrotoxicity. Our study provides a basis and rationale for the inhibition of cisplatin-induced nephrotoxicity by food-derived nutrients.

## Introduction

Cisplatin (CDDP), a commonly used chemotherapy drug in clinical practice, has a history of more than 170 years. CDDP is still used as an adjuvant drug in combination with radiotherapy or other drugs to improve tumor-inhibitory effects [1]. However, the clinical application of CDDP as a tumor suppressant is limited by its side effects; CDDP can cause nephrotoxicity, neurotoxicity, ototoxicity, and serious gastrointestinal reactions, and it may also produce drug resistance [2]. Acute kidney injury (AKI) induced by CDDP is a key factor limiting the clinical application of CDDP. The current clinical strategies to deal with CDDP-induced nephrotoxicity are mainly magnesium supplementation, hydration, and diuretic administration [3, 4]. However, large amounts of diuretic agents can subsequently cause dehydration, which can cause instability in patients with other underlying conditions [5]. Therefore, it would be an interesting strategy to suppress CDDP-induced nephrotoxicity through a food-derived route with functional ingredients without affecting body function.

Just as CDDP-induced apoptosis of cancer cells, CDDP-induced apoptosis of normal renal tubular epithelial cells is also strongly associated with oxidative stress. Almaghrabi et al. [6] demonstrated that CDDP induces oxidative stress in renal tissues by inhibiting free radical formation and the expression and activity of antioxidant enzymes. In addition, CDDP also reduces the intracellular levels of vitamin E, superoxide dismutase (SOD), vitamin C, and glutathione (GSH), and the reduction of antioxidant levels in the body will lead to oxidative stress [7, 8], which affects many signaling pathways that regulate cell growth, apoptosis, and tissue inflammation. It is conceivable that the use of antioxidants to antagonize CDDP-induced nephrotoxicity is a promising research direction. It has been reported that vitamin E [9], cyanidin [10, 11], ginseng [12, 13], and sinapic acid [14] show protective effects against CDDP-induced nephrotoxicity. However, the instability of many antioxidants makes their application a challenge. To overcome this issue, the encapsulation of molecules in nanostructures [15] and the use of metal chelation to modulate spatial resistance to enhance molecular stability [16] are feasible approaches. In the field of functional foods, it has become a trend to reduce the size of functional ingredients to the nano-level.

A large number of studies have shown that *Ganoderma Lucidum* spore oil (GLSO) is rich in triterpenoids, polysaccharides, and steroids. GLSO has antioxidative, antitumor, immune-enhancing, and inflammation-inhibitory effects, and it slows down cardiovascular and cerebrovascular diseases. Jiao et al. [17] demonstrated that GLSO inhibits tumor proliferation via the caspase pathway in vivo and in vitro. Moreover, compared with antibiotic treatment, GLSO treatment can significantly inhibit inflammation caused by burn and accelerate wound healing by regulating skin microbiota. In addition, GLSO can regulate macrophage phagocytosis and NK cell cytotoxicity in mice to enhance the body's immune function. Wu et al. [18] found that GLSO can induce the rearrangement of intestinal microorganisms and their metabolites and believed that the enhancement of immune function is highly correlated with the changes of related intestinal microorganisms. However, on the one hand, GLSO tastes bad and often causes discomfort when taken in liquid form due to its lipid solubility. On the other hand, since GLSO contains few other antioxidants and triterpenoids are prone to oxidation under photothermal conditions, rancidity affects the quality [19].

Selenium nanoparticles (SeNPs) have been reported to exhibit good bioavailability and exert strong anticancer and antioxidant effects. SeNPs are small and can be modified in a targeted manner [20, 21]. Li et al. [22] modified SeNPs with polysaccharides in order to improve the stability and bioactivity of polysaccharides and SeNPs; the in vivo results showed that polysaccharide-modified SeNPs reduced myocardial injury via the caspase, Bax/Bcl-2, and Fas/Fasl—pathways in a type 2 diabetes model. Meanwhile, SeNPs modified by coupling or surface modification showed stronger anticancer effects [23]. Chen et al. used adenosine triphosphate (ATP)-modified SeNPs to form ATP@SeNPs, which could induce apoptosis in cancer cells by increasing intracellular ROS levels through regulating caspase family enzyme activity [24].

Therefore, it was envisioned that the use of GLSO to modify SeNPs might be able to both prevent the premature acidification of GLSO and improve the stability of SeNPs, thus enhancing the stability and bioactivity of the overall nanosystem. Herein, GLSO extracted from a traditional Chinese medicine and nano-selenium, an emerging chemical material, were taken as the main research objects. GLSO was nano-treated, and then, the nano-GLSO (GLSO@NEs) was crosslinked with nano-selenium using Poloxam 407 solution. The GLSO-modified nano-selenium system (GLSO@SeNPs) was formed with small particle size and uniform dispersal. The stability and biological activity of spore oil and nano-selenium were improved, and the cytotoxicity and nephrotoxicity of CDDP were reduced, as shown by in vivo and in vitro experiments.

## Materials and methods

### Materials

GLSO was purchased from certain commercial company. Sodium selenite (Na_2_SeO_3_) and ascorbic acid (VC) were purchased from Sigma Aldrich (USA). PI, JC-1, and glutathione peroxidase (GSH-Px), SOD, malondialdehyde (MDA), CCK8, and BCA assay kits were obtained from Beyotime Biotechnology (China). Dulbecco’s modified eagle medium (DMEM), 1640 medium, penicillin–streptomycin, and fetal bovine serum (FBS) were purchased from Gibco (USA). Antibodies for flow cytometry were obtained from Biolegend (USA), and antibodies for Western blot (WB) and ELISA were obtained from Cell Signaling Technology (USA).

### Preparation and characterization of the nanosystems

To prepare GLSO@NEs, Tween-80 and anhydrous ethanol were mixed at room temperature for 5 min at 400 rpm. Next, 5 mL GLSO was gently added, followed by stirring for 5 min at room temperature at 800 rpm. Next, ultrapure water was slowly added and the volume was increased to 50 mL to create a 10% GLSO mixture. Next, 10% mixture of GLSO colostrum was transferred to a high-pressure homogenizer. The preliminary homogenization conditions were as follows: homogenization pressure, 200 bar for 2 min; loading frequency, 40 Hz. The high-pressure homogenization conditions were as follows: homogenization pressure, 900 bar for 10 min; injection frequency, 40 Hz. Finally, 10% of nano-sized GLSO@NEs was formed [25].

Next, 1 mL of 20 mM sodium selenite solution was mixed with 10 mg of mushroom polysaccharide in 3 mL of water, and 1 mL of 80 mM VC solution was gently added dropwise. The solution was stirred overnight (about 12 h). The SeNPs were collected by dialysis in a sink using 6000–8000 kDa dialysis bags for 24 h (water was changed every 12 h). The synthesized 1 mM SeNPs, 10% GLSO@NEs, and hydrogel were mixed at a ratio of 1:1:8 and then vortexed to form the GLSO@SeNPs (GLSO@NEs: 1% and SeNPs: 0.1 mM) [26].

### Characterization of GLSO@NEs, SeNPs, and GLSO@SeNPs

The hydrodynamic particle size and zeta potential of different nanosystems were measured by a Malvin particle size analyzer. After staining with phosphotungstic acid, the morphology and size of nanoparticles were observed by transmission electron microscopy (TEM) [27, 28].

### Stability assessment of GLSO@NEs, SeNPs and GLSO@SeNPs

The stability of GLSO@NEs, SeNPs and GLSO@SeNPs was assessed by monitoring their particle size and potential changes in cell culture medium (without phenolic red and FBS). Certain concentrations of the nanosystems were added to the medium and placed at 4 °C, and their particle size and potential information were measured at different time points (0, 12, 24, 48, 72, 168 h).

### Hemocompatibility of GLSO@NEs, SeNPs and GLSO@SeNPs

Healthy human-derived erythrocytes were used to demonstrate the good hemocompatibility of GLSO@NEs, SeNPs and GLSO@SeNPs. First, erythrocytes were diluted with PBS and aliquoted into test tubes and co-incubated with different nanosystems at 37 °C for a certain time (2 h, 4 h, 8 h). Meanwhile, a negative (PBS) and a positive (Triton X-100) control group were set up respectively, following by centrifugation to collect the supernatant for spectrophotometric detection (540 nm) to assess the hemolysis rate of nano-drugs. The hemolysis rate was calculated by the formula:

Hemolysis rate (%) = (λNanosystem − λNG)/(Λpg − λNG) × 100%. Where λNanosystem, λNG and λPG is the absorbance of nanoparticles, negative control and positive control group at 540 nm.

### Cell culture and CCK8 assay

Human kidney-2 (HK-2) cells, human hepatoma cells (HepG2 cells), and HeLa cells were purchased from American Type Culture Collection (ATCC, USA). HK-2 cells were cultured in 1640 medium, and cancer cells were cultured in DMEM. Media contained penicillin–streptomycin and 10% FBS. The cells were cultured in a humidified incubator (5% CO_2_ and 37 °C). To detect the effects of drugs on cell survival, 5000 cells/well were inoculated in 96-well plates and cultured for 24 h. Nanosystem drugs were added to pre-protect the cells for 2 h, followed by the addition of CDDP for 24 h. Subsequently, 100 μL/well of CCK8 solution was added, and the cells were incubated at 37 °C for 2 h. Absorbance values were measured at 450 nm. The viability of cells relative to control cells was calculated [29].

### Flow cytometric analysis

PI was used to analyze the proportions of HK-2 cells in different phases of the cell cycle as previously described [25, 26]. Briefly, HK-2 cells were pretreated with nanosystem drugs for 2 h and then treated with or without CDDP (4 μg/mL) for 24 h. Cells were collected, washed with cold PBS, and then immobilized overnight with 70% ethanol. After washing with PBS twice, PI staining was performed at 4 °C for 30 min, and flow cytometry (Beckman Coulter) analysis was conducted. Multi-cycle software was used for data analysis. For each experiment, 10,000 events were recorded per sample. Flow cytometry data were analyzed using modified cytexpert software.

### Caspase activity assay

HK-2 cells (1 ×10^5^ cells per well, 5 mL) were cultured in 6-cm dishes. Cells were treated with nanosystem drugs and CDDP, incubated for 24 h, and collected. After lysis, the protein concentration was detected using a BCA kit. Then, 100 μg protein/well and 5 μL specific caspase 3 substrate were added to a black 96-well plate. After incubation at 37 °C for 2 h, the fluorescence intensity of activated caspase 3 was detected at λ_ex_ = 360 nm and λ_em_ = 460 nm. The fluorescence intensity of activated caspase 8/9 was measured at λ_ex_ = 351 nm and λ_em_ = 430 nm.

### Evaluation of ROS generation

HK-2 cells were treated with nanosystem drugs for 2 h, incubated with DCFH-DA for 30 min, and washed with PBS twice. Then, CDDP was added and the fluorescence values were immediately detected at λ_ex_ = 488 nm and λ_em_ = 525 nm.

### Mitochondrial morphology analysis

HK-2 cells (1 ×10^5^ cells per well, 2 mL) were cultured in 1.5 cm dishes. The cells were treated with nanosystem drugs and CDDP as described above, followed by Mito-Tracker (Green) and H33342 (blue) staining. Cells were washed with PBS twice. Mitochondria were photographed using a confocal laser scanning microscope (LSM 880 with AiryScan, Carl Zeiss) [30]. Fluorescence images were analyzed using Zen Blue Software.

### Mitochondrial membrane potential analysis

The mitochondrial membrane potential (MMP) was analyzed using JC-1 as previously described [31]. In brief, HK-2 cells (1 ×10^5^ cells per well, 5 mL) were cultured in 6-cm dishes. The treated cells were collected and stained with JC-1 (10 μg/mL) for 10 min. Stained cells were immediately detected by flow cytometry (Beckman Coulter). Flow cytometry data were analyzed using modified cytexpert software.

### Western blot analysis

The effects of CDDP and nanosystem drugs on the protein expression of caspases, MAPK-related proteins, and AKT were analyzed by WB [32]. Total cellular proteins were extracted in lysis buffer, and protein samples were boiled in PBS with loading buffer, separated by SDS-PAGE, and transferred to a membrane, which was incubated with specific antibodies to detect the proteins of interest. Protein bands were imaged and analyzed using clinx software. WB grayscale images were analyzed using clinx chemical analysis software.

### Nephrotoxicity analysis in vivo

Male 6 week-old C57/BL6J mice (22–24 g) were purchased from Jicui Yaokang company (Guangdong, China). All animal experiments were approved by the Ethics Committee of Jinan University (Approval No. IACUC-20210429-04). The mice were housed in the animal house of Jinan University with a 12/12 h day/night cycle at 22–26 °C with a relative humidity of 60–80%. The mice had free access to food and water. The mice were acclimatized for 2 weeks before the experiments started. The mice were divided into seven groups: (i) the control group, (ii) the CDDP group (15 mg/kg), (iii) the CDDP with GLSO@NEs (5 mL/kg·bw) group, (iv) the CDDP with GLSO@SeNPs (Se 2 mg/kg·bw) group, (v) the CDDP with GLSO@SeNPs (Se 4 mg/kg·bw) group, (vi) the CDDP with SeNPs (Se 4 mg/kg·bw) group, and (vii) the GLSO@SeNPs (Se 4 mg/kg·bw) group. The mice were gavaged with nanosystem drugs or saline for 7 consecutive days. CDDP (15 mg/kg, i.p.) was administered on the fourth day. Mice were killed on the eighth day, and the serum, kidney, and spleen were collected. Renal function was evaluated by measuring serum creatinine (CRE) and blood urea nitrogen (BUN) levels in the serum.

A portion of kidney tissue was weighed for the detection of renal oxidative stress status (SOD, MDA, GSH). Additionally, kidney tissues were subjected to determination of selenium morphology using protease enzymatic lysis, followed by filtration using HPLC-ICP-MS [33]. In addition, renal histopathological sections were analyzed. Fluorescence images were analyzed using Zen Blue Software.

### Flow cytometry analysis of immune cells

The analysis of immune cell proliferation was conducted following previously reported methods [34]. The freshly obtained spleen and kidney were homogenized, lysed, and cleaned with PBS, and the cells were stained on the surface and intracellularly with antibodies targeting CD45, CD3, CD8, CD4, CD25, FoxP3, NK1.1, NKp46, CD11b, Ly6G/Ly6C, F4/80, and CD206. Treated cells were analyzed by flow cytometry. Flow cytometry data were analyzed using modified cytexpert software.

### Statistical analysis

SPSS 19.0 and GraphPad prism 8.0 were used for statistical analysis. Graphs were prepared using GraphPad prism 8.0. Data are expressed as mean ± standard error of the mean (SEM). Differences between multiple groups were analyzed by one-way ANOVA. *P < 0.05 was considered statistically significant, and **P < 0.01 indicated a highly significant difference.

## Results and discussion

### In vitro antioxidant and nephroprotective activity of Se@TE NPs

**Fig. 1:**
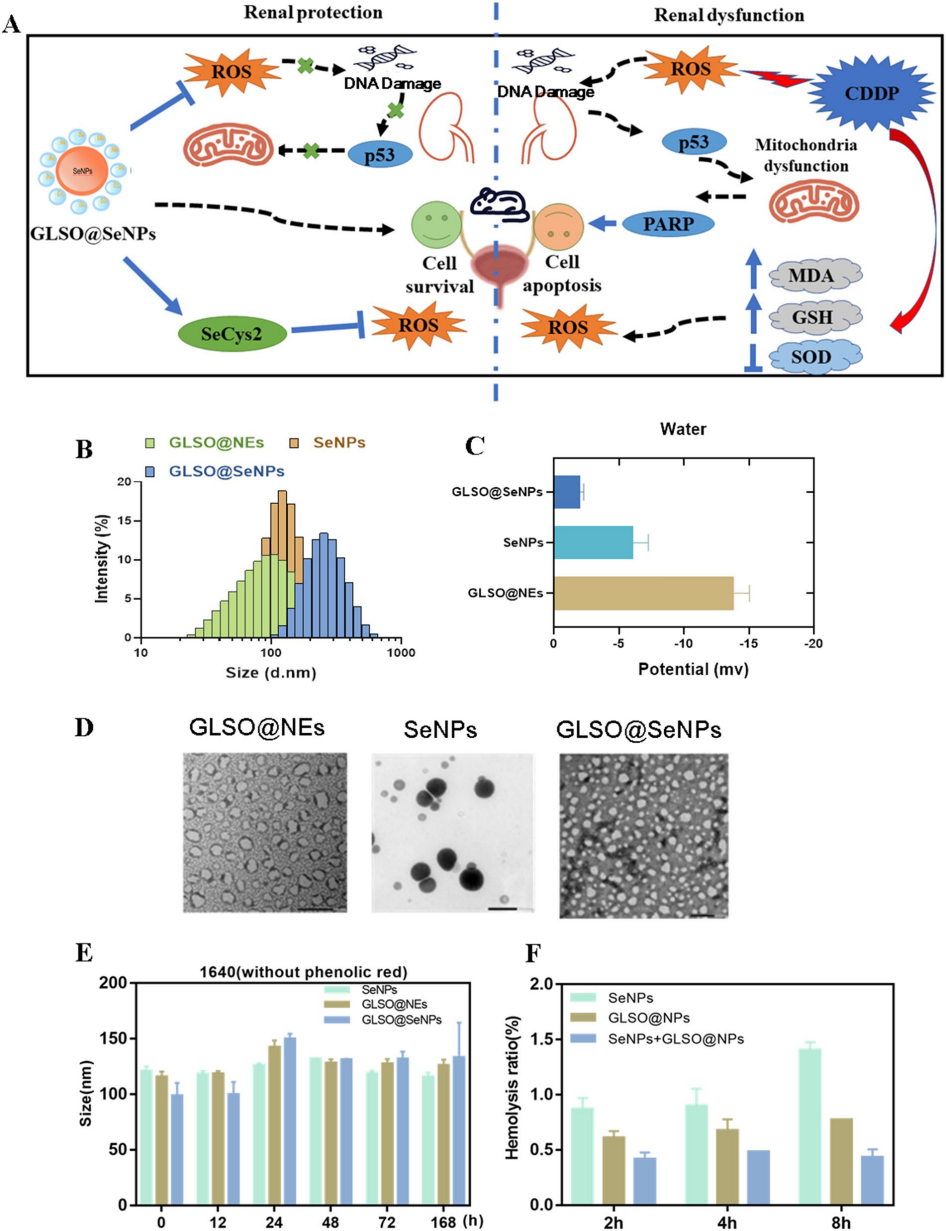
Characterization of nanosystems. **A** Protective effects of GLSO@SeNPs against CDDP-induced nephrotoxicity. **B** Particle size of three groups of nanosystems. **C** Zeta potential of three groups of nanosystems. **D** TEM images of nanosystems. **E** The particle size of nanosystems in 1640 (without Phenolic red) in 168 h. **F** Hemolysis percentage of nanosystems

Three drugs were synthesized in this study (**‘‘**Preparation and characterization ofthe nanosystems**’’** Sect). As shown in Fig. 1B–D, the sizes of GLSO@NEs, SeNPs, and GLSO@SeNPs were 100 nm, 126 nm, and 230 nm, respectively, as determined using a Malvern particle sizer. The TEM results further showed that the particles are nano-sized and uniformly distributed. The negative surface zeta potentials further support this conclusion. Moreover, the particle size values of nanosystems in phenol red-free culture medium were basically stable at about 100–150 nm with time. The above experimental results indicate that the synthesized nanosystems have a satisfactory stability (Fig. 1E). After homogenization treatment, nano-sized drugs were found to have a larger relative specific surface area; they were more efficiently absorbed by the gastrointestinal wall villi, and they significantly improved spore oil solubility [35]. Further, as shown in Fig. 1F, nanomedicines all exhibited less than 1.5% hemolysis after 8 h Coincubation with RBCs.

**Fig. 2:**
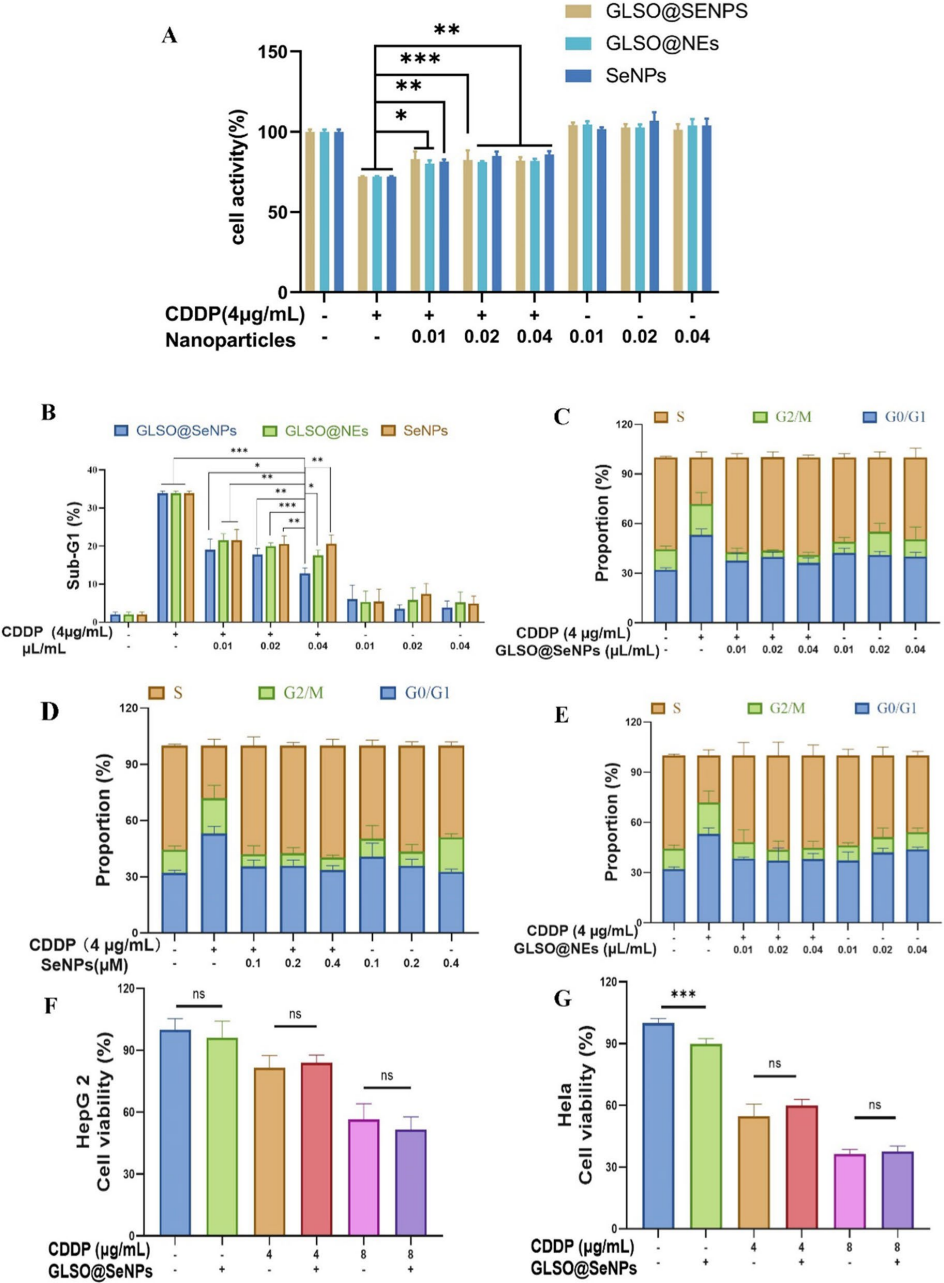
Protective effects of nanosystems. **A** Effect of different concentrations of nanoparticles on the cytotoxicity of CDDP. **B** Nanosystems significantly decreased the proportion of sub-G1 cells after CDDP treatment. **C**–**E** Nanosystems alleviated the cell cycle arrest induced by CDDP. **F**, **G** GLSO@SeNPs did not impair the inhibitory activity of CDDP on HepG2 and HeLa cells. *P < 0.05, **P < 0.01, ***P < 0.001; ns, not significant, one-way ANOVA

The data in Fig. 2A showed that different concentrations of nanoparticles were effective in protecting against cisplatin-induced cytotoxicity and that the nanoparticles alone did not have toxic effects on cells. As shown in Fig. 2D–F, CDDP significantly arrested the cell cycle of HK-2 cells in the G0/G1 phase; the proportion of HK-2 cells in the G0/G1 phase in the CDDP group was 53.14%, while the corresponding proportion in the control group was only 32.04%. After treatment with different concentrations of nanosystem drugs, the G0/G1 phase arrest was significantly alleviated, whereas treatment with nanosystem drugs alone did not significantly affect the HK-2 cell cycle distribution. However, as shown in Fig. 2C, CDDP caused a significant surge in the proportion of cells in the sub-G1 phase (33.88%), and after pretreatment with different concentrations of nanosystem drugs, the proportions of cells in the sub-G1 phase were decreased to various degrees; for example, 0.04 μL/mL GLSO@SeNPs significantly reduced the proportion of sub-G1 cells to 12.83%, which showed the best protective effect in terms of reducing the proportion of apoptotic cells, with significant differences compared to the other nanosystem drug groups.

The cell cycle of normal cells is an orderly event regulated by the cyclin family and the cyclin-dependent kinase (CDK) family. External stimuli can block the cell cycle in a certain phase, thereby leading to DNA damage [36] and inhibiting cell proliferation [37]. To investigate the effects of the nanosystems on CDDP-induced apoptosis and cell proliferation, PI staining was applied to determine the proportions of cells in different cell cycle phases based on the amount of DNA detected in the cells. In general, CDDP induced cell cycle arrest, and nanosystem drugs restored the cell cycle [38].

Considering that the inhibitory effect of CDDP on tumor cells is also largely related to a series of pathways activated by ROS, we verified whether the antioxidant capacity and free radical scavenging function of the nanosystems itself affect the anticancer effects of CDDP. When cancer cells were treated with both CDDP and 0.04 μL/mL GLSO@SeNPs, the cell survival rate of HepG2 cells was not significantly different from the survival rate after treatment with CDDP only (Fig. 2G). Notably, treatment with GLSO@SeNPs alone also significantly reduced the survival rate of HeLa cells (Fig. 2H). At the cellular level, this verifies that treatment with GLSO@SeNPs does not inhibit the anticancer effects of CDDP, and treatment with GLSO@SeNPs also inhibits the proliferation of HeLa cells.

### GLSO@SeNPs attenuate CDDP-induced apoptosis by inhibiting caspase activation

**Fig. 3:**
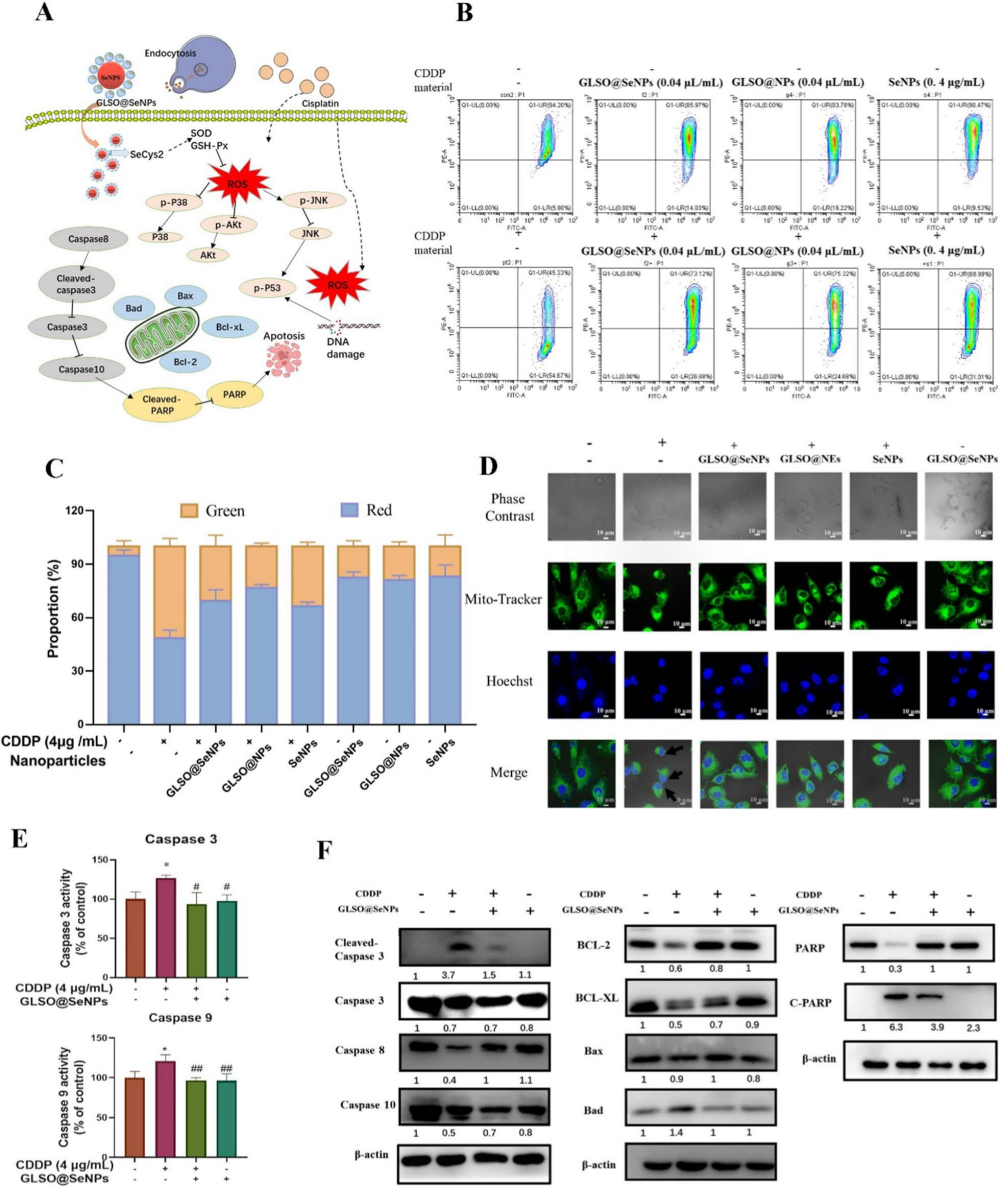
**A** GLSO@SeNPs inhibit CDDP-induced caspase-regulated mitochondrial damage. **B** MMP and mitochondrial morphology in HK-2 cells. **C** Quantitative analysis of JC-1 intensity proportion for flow cytometry analysis in (**a**). **D** mitochondrial morphology in HK-2 cells. **E** Activity of caspases 3/9 was evaluated with specific fluorescent substrates. **F** HK-2 cells were treated with 0.04 μL/mL GLSO@SeNPs with 4 μg/mL CDDP, and then, intracellular protein expression levels of caspase 3/8/10, Bcl-2, Bcl-xL, Bad, Bax, and PARP were analyzed. *P < 0.05, ^#^*P* < 0.05, ^##^*P* < 0.01; *ns* not significant; one-way ANOVA

CDDP significantly downregulated Bcl-2 and Bcl-xL protein levels, which were also significantly suppressed in the GLSO@SeNPs group. With respect to Bax and Bad, CDDP did not cause a significant increase in Bax protein levels, and CDDP only caused a small increase in Bad levels relative to the control group (Fig. 3F).

Abnormal expression of the Bcl-2 protein family induces a decrease in MMP and induces mitochondrial membrane permeabilization, resulting in the release of large amounts of Cyt *c* and Fas from mitochondria into the cytoplasm, which promotes the assembly of apoptotic vesicles and activates the caspase family of proteins, such as caspase 3, caspase 8, and caspase 9, causing a cascade reaction and further activating other caspase proteins, including caspase 10 [39]. Activated caspase 3 can cleave and degrade PARP, in addition to activating histones and thereby regulating the ATR pathway, which are associated with DNA repair and gene translation, thus arresting DNA repair and initiating DNA degradation [40].

As shown in Fig. 3F, CDDP induced the activation of caspase 3, caspase 8, caspase 9, and caspase 10, as indicated by the fact that the protein levels of cleaved caspase 3 were dramatically increased in the CDDP group. After pretreatment with GLSO@SeNPs, the protein levels of caspase 3, caspase 8, and caspase 10 were decreased to various degrees, indicating that the domains of caspase 3, caspase 8, and caspase 10 were cleaved and activated, significantly inhibiting the activation of caspase 3, caspase 8, and caspase 10. These observations were confirmed by ELISA experiments (Fig. 3E). Furthermore, after CDDP treatment, intracellular PARP protein levels were dramatically downregulated, whereas cleaved PARP levels were significantly upregulated. Pretreatment with GLSO@SeNPs significantly inhibited PARP activation.

The most direct result of mitochondrial damage is mitochondrial dysfunction, leading to reduced ATP synthesis and the release of large amounts of proapoptotic factors, thus accelerating the apoptotic process [41]. Mitochondrial damage is manifested by a decrease in MMP, disruption of the mitochondrial structure [42], and a decrease in the number of mitochondria in HK-2 cells. The MMP was measured using a JC-1 kit. As shown in Fig. 3B, C, CDDP significantly reduced the red fluorescence of HK-2 cells relative to the control group, and pretreatment with high concentrations of GLSO@NEs, SeNPs, and GLSO@SeNPs could significantly reverse this change, indicating that pretreatment with nanosystem drugs can effectively inhibit the CDDP-induced loss of MMP. Moreover, morphological changes of mitochondria are also an important indicator of mitochondrial damage. As shown in Fig. 3D, CDDP treatment caused the fragmentation of mitochondria (as shown by the arrow); in addition, the nucleus also underwent significant wrinkling and partial fragmentation. At high concentrations, nanosystem drugs could significantly inhibit the CDDP-induced structural damage of mitochondria; in the nanosystem drug group, more intact and normal spindle-shaped mitochondria were observed.

### GLSO@SeNPs inhibit apoptosis by suppressing the ROS-induced MAPK pathway

**Fig. 4:**
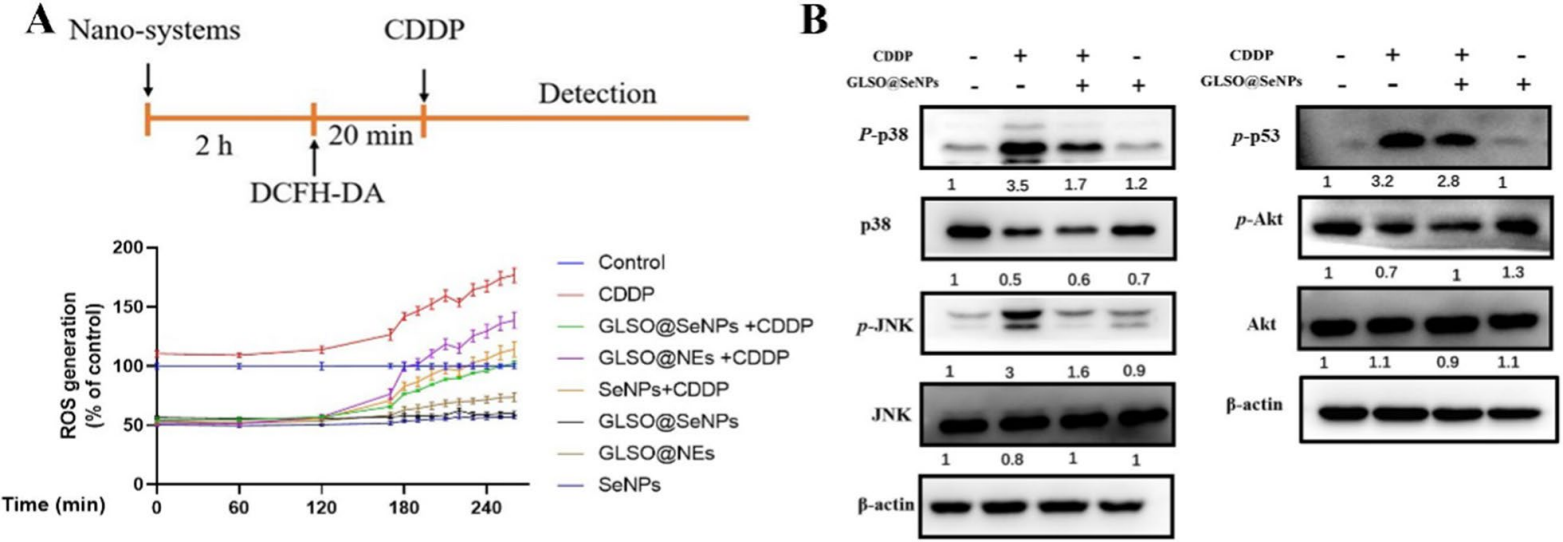
GLSO@SeNPs decreased the CDDP-induced oxidation levels and inhibited the MAPK signaling pathway in HK-2 cells. **A** Nanosystem drugs suppressed CDDP-induced ROS accumulation in HK-2 cells, as determined by DCFH-DA staining. **B** GLSO@SeNPs inhibited apoptosis by suppressing MAPK-related pathways

CDDP-induced apoptosis in HK-2 cells is mediated by the mitochondrial oxidative stress pathway, the endoplasmic reticulum stress pathway, and the death receptor pathway. All three are associated with intracellular ROS levels, and ROS accumulation is related to the production of proapoptotic factors after organelle damage [43], which is a key part of CDDP-induced damage in HK-2 cells. We aimed to make use of the strong antioxidant properties and ROS scavenging ability of spore oil and SeNPs to antagonize the CDDP-induced ROS accumulation and stop the process of apoptosis upstream of the CDDP-induced apoptosis pathway. As shown in Fig. 4A, relative to the control group, the ROS levels of HK-2 cells started to rise slowly after 1 h of CDDP treatment and rapidly after 3 h; pretreatment with high concentrations of GLSO@NEs, SeNPs, and GLSO@SeNPs significantly reduced the CDDP-induced ROS accumulation. Interestingly, treatment with nanosystem drugs alone at high concentrations significantly reduced the ROS levels in HK-2 cells. GLSO@SeNPs had a stronger ROS scavenging ability than SeNPs and GLSO@NEs, which again demonstrated that GLSO@SeNPs could exert stronger cytoprotective effects than GLSO@NEs and SeNPs alone.

The WB results showed that CDDP treatment induced p38 phosphorylation in HK-2 cells relative to the control group. In HK-2 cells pretreated with GLSO@SeNPs, the p38 phosphorylation level was significantly downregulated (Fig. 4B). In addition, CDDP significantly increased the phosphorylation level of JNK, while in the CDDP + GLSO@SeNPs group, the phosphorylation level of JNK was reduced to control levels. Furthermore, total JNK protein expression did not differ significantly between the groups (Fig. 4B). CDDP treatment induced a significant increase in the intracellular p53 phosphorylation level relative to the control group, whereas in the CDDP + GLSO@SeNPs group, the p53 phosphorylation level was reduced to some extent compared with the CDDP group. Phosphorylation of AKT protein can inhibit the process of apoptosis. Therefore, we examined the phosphorylation level of AKT by WB. As shown in Fig. 4B, the phosphorylation level of AKT in the CDDP group was significantly elevated relative to the control group; nanosystem drugs significantly decreased AKT phosphorylation to control levels. AKT protein expression was not significantly different between the groups.

### GLSO@SeNPs reduce CDDP-induced nephrotoxicity in vivo

**Fig. 5:**
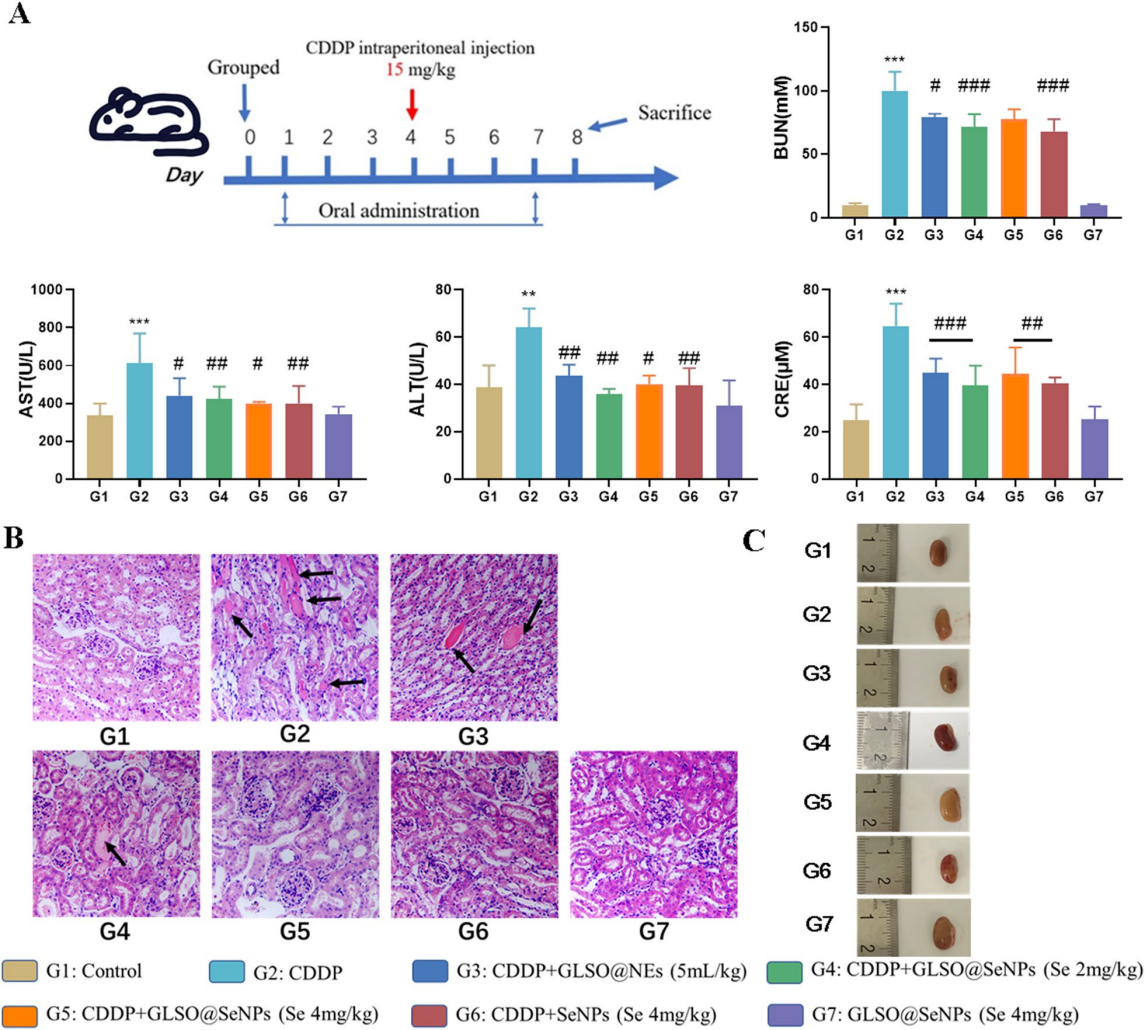
GLSO@SeNPs reduced CDDP-induced nephrotoxicity in vivo. **A** Blood urea nitrogen (BUN) and creatinine (CREA) are indicative of renal injury; aspartate aminotransferase (AST) and alanine aminotransferase (ALT) are indicative of liver injury. **B** Histopathological analysis of kidney sections. **C** Mouse kidney

To verify the protective effect of the nanosystems against CDDP-induced nephrotoxicity in vivo, a mouse nephrotoxicity model was established (Fig. 5A) as previously described [44, 45]. As shown in Fig. 5A, the serum levels of BUN and CRE, which serve as indicators of renal injury, were significantly elevated in C57 mice treated with CDDP (15 mg/kg, i.p.), while in mice treated with both CDDP and nanosystem drugs, these levels decreased to different degrees. In addition, the BUN and CRE levels of mice treated with high doses of GLSO@SeNPs alone were not significantly different from those of thecontrol group. Furthermore, it is known that in a nephrotoxicity mouse model, CDDP can induce hepatotoxicity in addition to renal injury, and the severity of hepatotoxicity is positively correlated with the injected dose of CDDP [46]. As shown in Fig. 5A, CDDP treatment caused a significant elevation of ALT and AST serum levels, while mice that also received different concentrations of nanosystem drugs showed a significant decrease in ALT and AST levels. These results reveal that GLSO@NEs, SeNPs, and GLSO@SeNPs can protect against CDDP-induced AKI and liver injury.

Histopathological analysis provides a visual basis for diagnosing whether or not a tissue is diseased. The histological structure and pathology of the kidney sections were determined by HE staining. As shown in Fig. 5B, the renal tubules in the control group were clearly structured with no obvious abnormalities. In the mice treated with CDDP, the tubules in the kidney were damaged, with a large number of protein tubular patterns (shown by arrows) in the tubular lumen, and some of the renal epithelial cells were necrotic and detached. In the CDDP-treated mice gavaged with GLSO@NEs and low-dose GLSO@SeNPs, some protein tubular patterns were still visible in the kidneys, but the number of protein tubular patterns was significantly improved, the number of necrotic cells was significantly reduced, and tubular damage was inhibited. In the CDDP-treated mice gavaged with high-dose GLSO@SeNPs and SeNPs, no obvious protein tubular patterns were observed in the kidney sections, no inflammatory cell infiltration was found, and the tubular and glomerular structures were close to normal. In the CDDP-treated mice gavaged with high-dose GLSO@SeNPs and SeNPs, no obvious protein tubular patterns were observed in the kidney sections, no inflammatory cell infiltration was found, and the tubular and glomerular structures were close to normal. The morphological picture of the kidney tissues showed that the normal mouse kidney tissues were bean-shaped, smooth and vascularized, while after CDDP injury, the kidney appeared to be sagging and deformed, and the rupture of the renal peritoneum and the rupture of the renal tip vessels could be seen, which caused obvious hematuria with extravasation of urine. In Fig. 5C, the kidneys in the GLSO@NEs-protected group regained their normal bean shape, and the kidneys in the low and high doses of GLSO@SeNPs-protected groups were full of blood and clear in outline. The kidneys in the SeNPs group had normal morphology but were slightly smaller, probably related to the difference in the mice's own body weight, while the kidneys in the GLSO@SeNPs-treated group alone were morphologically similar to the control group, with no pathological features.

### GLSO@SeNPs reduce CDDP-induced renal oxidative damage

As confirmed in the previous cellular experiments, the nanosystem drugs could significantly alleviate CDDP-induced ROS accumulation and thus oxidative stress in vitro. To investigate the effects of the nanosystem drugs on oxidative stress in mouse kidney tissues, we analyzed several conventional oxidative stress indicators.

**Fig. 6:**
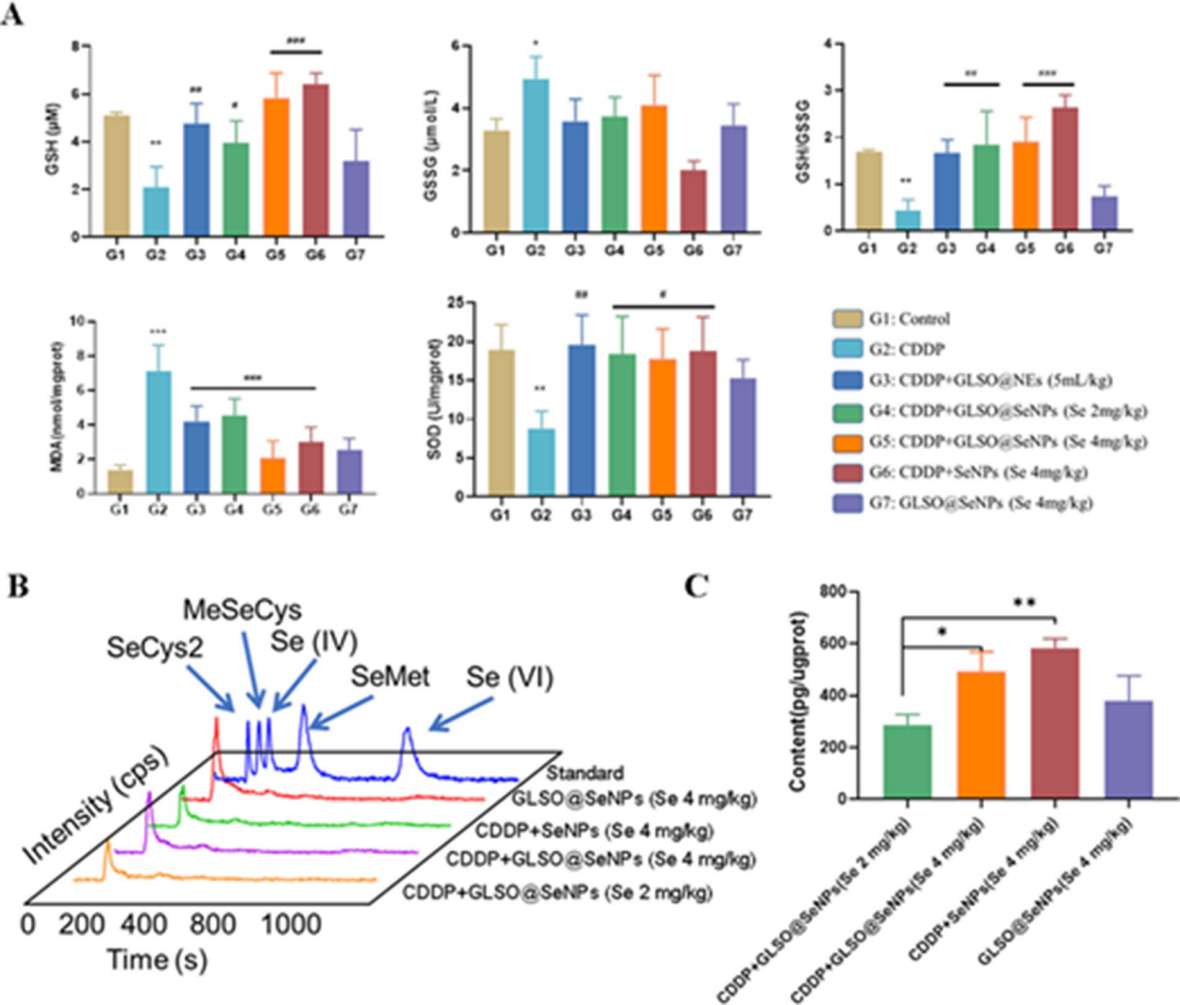
GLSO@SeNPs reduced CDDP-induced renal oxidative damage. **A** GSH, SOD, and MDA levels in kidney tissues. **B**, **C** Se-Cys2 levels were quantified in mouse kidney tissues by HPLC. *P < 0.05, **P < 0.01, ***P < 0.001; one-way ANOVA

As shown in Fig. 6B, CDDP depleted GSH and significantly reduced the GSH/GSSG ratio in the mouse kidney, while the GSH level and the GSH/GSSG ratio in the kidneys of mice treated with nanosystem drugs were significantly increased compared with the CDDP group. In addition, as shown in Fig. 6B, the MDA levels in the kidneys of CDDP-treated mice were significantly elevated, and the nanosystem drugs significantly inhibited this elevation. CDDP strongly reduced SOD levels in the kidney tissues, and pretreatment with nanosystem drugs significantly inhibited the decrease in SOD levels (Fig. 6B). These results suggest that different doses of GLSO@NEs, SeNPs, and GLSO@SeNPs can protect against CDDP-induced oxidative stress and maintain oxidative homeostasis.

Neutral selenium atoms do not directly exert their biological activity in the organism; selenium needs to be bioconverted to other valent forms by enzymes in the body to perform its functions such as inhibition of migration and proliferation of cancer cells and antioxidant effects [47]. The main active forms of selenium in the organism are selenocysteine (SeCys2) and selenomethionine (Se-Met) [48]. Most of the identified selenoproteins have been reported to be oxidoreductases, involved in the maintenance of oxidative homeostasis. SeCys2 is abundantly present in GSH-Px and thioredoxin reductase, showing antioxidant activity due to its location in the catalytic active site, and its anticancer efficacy is associated with the p53-regulated DNA damage pathway, the Bcl-2 protein family [49], and unfolded protein effects [50]. As shown in Fig. 6C, GLSO@SeNPs and SeNPs were mainly converted to SeCys2 in the mouse kidney, and after CDDP injection and treatment with GLSO@SeNPs and SeNPs, the mouse kidneys showed a dose-dependent change in SeCys2 levels (Fig. 6D). Therefore, GLSO@SeNPs and SeNPs were bioconverted from the zero-valent state of SeNPs to SeCys2 in mice, which inhibited CDDP-induced nephrotoxicity.

### GLSO@SeNPs regulate immune cells in kidney and spleen tissues

It has been demonstrated that SeCys2 can play a regulatory role in the immune system response [51], and the regulatory effects of GLSO on NK cells and macrophages have been reported in the literature [52, 53]. We investigated the effects of nanosystem drugs on CDDP-induced inflammation and explored their regulatory effects on immune cells. Among immune cells, T lymphocyte subsets are important indicators of the body's immune function; specifically, CD3^+^CD4^+^ T cells, CD3^+^CD8^+^ T cells, and regulatory T cells (Tregs) play important functions in humoral and cellular immunity. CD3^+^CD4^+^ T cells and CD3^+^CD8^+^ T cells are clinically sensitive indicators reflecting the strength of the body's immune system. CD3^+^CD4^+^ T cells are T helper cells, while CD3^+^CD8^+^ T cells are the most lethal T cells; both are important immune cells in the human immune system [54]. While the mechanism of immunosuppression in CD4^+^FOXP3^+^ cells (Tregs) is not clear, some articles suggest that their suppressive function may be related to C–C chemokine receptor type 4 (CCR4), which is abundantly expressed on their surface [55]. In addition, MDSCs can indirectly perform immunosuppressive functions by secreting arginase 1 (ARG-1), inducible nitric oxide synthase (iNOS), and ROS to suppress T lymphocytes and simultaneously induce differentiation to produce Tregs [56, 57]. In addition, macrophages are highly plastic and can be polarized into M1 and M2 macrophages, depending on the stress conditions of the body. M1 and M2 macrophages exhibit completely opposite immunomodulatory effects. M1 macrophages are proinflammatory macrophages induced by lipopolysaccharides (LPSs) and interferon-γ (IFN-γ), which can release large amounts of proinflammatory factors [58]. M2 macrophages are induced by cytokines such as IL-4, IL-10, and TGF-β. Polarization between M1 and M2 macrophages is mutually reversible, and macrophage polarization from M1 to M2 or the other way around is a topic of interest in the study of the anti-inflammatory and antitumor immune system [59].

**Fig. 7:**
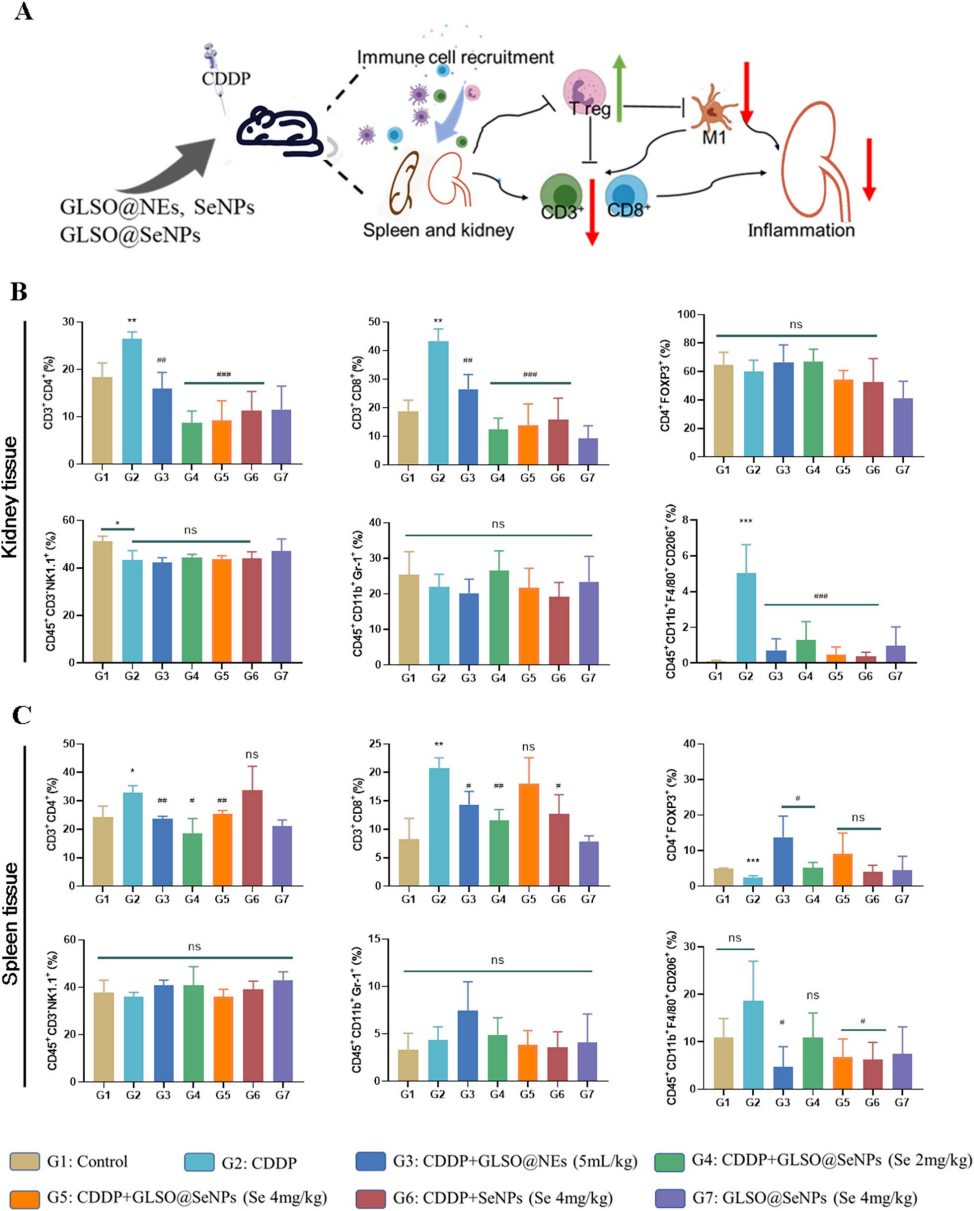
**A** GLSO@SeNPs regulated immune cells in kidney and spleen tissues. **B**, **C** Flow cytometry analysis of immune cells in kidney and spleen of mice. *P < 0.05, **P < 0.01, ***P < 0.001, ^#^*P* < 0.05, ^##^*P* < 0.01, ^###^*P* < 0.001, compared with the G1 and G2 groups in corresponding comparisons; ns, not significant, compared with the G2 group in corresponding comparisons; one-way ANOVA

As shown in Fig. 7, in both spleen and kidney tissues, the proportions of CD3^+^CD4^+^ T cells and CD3^+^CD8^+^ T cells were significantly increased by CDDP, while treatment with nanosystem drugs reversed the CDDP-induced changes; in addition, in the spleen, CDDP significantly reduced Treg counts, and similarly, treatment with nanosystem drugs, especially GLSO@NEs, increased the proportion of CD4^+^FOXP3^+^ cells to varying degrees; however, the proportion of CD4^+^FOXP3^+^ cells in kidney tissues showed no significant difference between the control, CDDP, and intervention groups. In addition, the proportion of CD45^+^CD3^−^NK1.1^+^ cells (NK cells) in spleen tissues did not differ significantly between groups, but in kidney tissues, CDDP significantly reduced the proportion of CD45 + CD3^−^NK1.1 + cells, and treatment with nanosystem drugs did not significantly reverse this change. With respect to macrophages, the proportions of CD45^+^CD11b^+^GR-1^+^ cells (MDSCs) were not significantly different in spleen and kidney tissues between groups. In addition, the proportion of CD11b^+^F4/80^+^CD206^+^ cells (M1 macrophages) tended to increase to some extent in the spleen of the modeling group and decreased to varying degrees upon treatment with nanosystem drugs. Similarly, in kidney tissues, the proportion of M1 macrophages, which was increased by CDDP, was significantly reduced upon treatment with nanosystem drugs.

## Conclusion

As a first-generation platinum-based anticancer chemotherapy drug, CDDP is widely used in the treatment of a variety of solid tumors, including genitourinary tumors, head and neck cancers, and leukemia. The main mechanism by which CDDP inhibits cancer is hydrolysis, generating nucleophilic groups to damage cancer cell DNA and thus inhibiting tumor proliferation. However, CDDP does not specifically bind to cancer cells and therefore causes tissue damage and a wide range of side effects, such as nephrotoxicity, ototoxicity, and neurotoxicity, which greatly limits the clinical application of CDDP [45]. Because of the excretory and enrichment functions of the kidney, the concentration of CDDP in the renal tubules is five times higher than that in the blood. CDDP entering the renal epithelium can cause renal impairment by generating large amounts of ROS, and most of the molecular signaling pathways that are subsequently activated revolve around ROS [44].

It has been shown that the main mechanism by which CDDP causes oxidative stress is through the production of large amounts of ROS and NOS. Large amounts of oxygen radicals can damage membrane proteins and thus alter mitochondrial membrane permeability and membrane lipid fluidity, which, on the one hand, can alter the antioxidant function of mitochondria and result in the release of large amounts of ROS and, on the other hand, can provoke the peroxidation of unsaturated fatty acids in biological membranes and result in the production of large amounts of toxic products such as MDA, which can disrupt the oxidative homeostasis of the organism[60]. We found that GLSO@SeNPs inhibit the mitochondrial apoptotic pathway by modulating the ROS-mediated MAPK pathway, inhibiting the caspase protein cascade, and interfering with the AKT signaling pathway, inhibiting the secretion of inflammatory factors and in turn inhibiting apoptosis in HK-2 cells, as shown in in vitro experiments.

Herein, we established a CDDP-induced AKI mouse model, investigated the beneficial effects of different doses of GLSO@NEs, GLSO@SeNPs, and SeNPs on CDDP-induced kidney injury in vivo, and examined the underlying mechanisms. We found that the nanosystem drugs can alleviate CDDP-induced nephrotoxicity by modulating oxidative stress in kidney tissues. We also investigated the in vivo biotransformation of selenium and confirmed that GLSO@SeNPs and SeNPs can be converted to SeCys2 in vivo, which may play a protective role.

In summary, different dosages of GLSO@NEs, GLSO@SeNPs, and SeNPs administered by gavage could effectively modulate the CDDP-induced inflammatory response. The effects on the inflammatory response were mainly derived from their regulatory effects on the proportions of immune cells in kidney and spleen tissues, that is, the proportions of CD3^+^CD4^+^ T cells, CD3^+^CD8^+^ T cells, and M1 macrophages in kidney and spleen tissues were reduced, and the proportion of anti-inflammatory Tregs was increased.
